# Murciano-Granadina Goat Performance and Methane Emission after Replacing Barley Grain with Fibrous By-Products

**DOI:** 10.1371/journal.pone.0151215

**Published:** 2016-03-16

**Authors:** Carla Ibáñez, Patricia Criscioni, Haritz Arriaga, Pilar Merino, Francisco Juan Espinós, Carlos Fernández

**Affiliations:** 1 Facultad de Veterinaria y Ciencias Experimentales, Departamento de Producción Animal y Salud Pública, Universidad Católica de Valencia, 46001, Valencia, Spain; 2 Research Centre ACUMA, Animal Science Department, Polytechnic University of Valencia, 46022, Valencia, Spain; 3 NEIKER-Tecnalia, Environment Quality Department, Bizkaia Technology Park, p. 812. 48160, Derio, Bizkaia, Spain; National Institute of Agronomic Research, FRANCE

## Abstract

The aim of this experiment was to study the effects of substituting dietary barley grain with orange pulp or soybean hulls on energy, nitrogen and carbon balance, methane emission and milk performance in dairy goats. Twelve Murciano-Granadina dairy goats in midlactation were selected and divided into three groups based on similar body weight (42.1 ± 1.2 kg) and milk yield (2.16 ± 0.060 kg/goat/day). The experiment was conducted in an incomplete crossover design where one group of four goats was fed a mixed ration of barley grain (BRL), another group of four goats replaced barley grain with orange pulp (OP) and the last group of four goats with soybean hulls (SH). After adaptation to diets, the goats were allocated to individual metabolism cages and intake, faeces, urine and milk were recorded and analysed. Then, gas exchange measurements were recorded by a mobile open-circuit indirect calorimetry system using a head box. Dry matter intake was similar for all three groups (2.03 kg/d, on average). No influence of the diet was observed for energy balance and the efficiency of use of metabolizable energy for milk production was 0.61. The OP and SH diets showed greater (P < 0.05) fat mobilization (-42.8 kJ/kg of BW^0.75^, on average) than BRL (19.2 kJ/kg of BW^0.75^). Pentadecanoic acid (15:0) and heptadecanoic acid (17:0) were potential biomarkers of rumen function because the higher contents found in the milk of OP and SH goats than BRL suggest a negative impact of these diets on rumen bacterial metabolism; probably linked to the lower nitrogen supply of diet OP to synthesize microbial protein and greater content of fat in diet SH. Replacement of cereal grain with fibrous by-products did not increased enteric methane emissions (54.7 L/goat per day, on average). Therefore, lactating goats could utilize dry orange pulp and soybean hulls diets with no detrimental effect on milk performance.

## Introduction

To achieve maximum milk production potential by means acceptable to consumers, feeding systems for dairy ruminants need to ensure high energy intake, among other factors. This might be accomplished by raising the dietary concentration of rapidly degraded non-fibrous carbohydrates (NFC), such as starch from cereal grain. Increasing the concentration of NFC in diets for dairy ruminants, however, can lead to undesirable ruminal fermentation, compromising the nutrient supply for production of milk and milk components. The partial replacement of cereal grain with low starch by-product feeds represents a potential alternative to overcome this limitation. By-products from agriculture may be of interest not only for reducing feeding cost but also to minimize environmental problems associated with side-effect accumulation [1].

Recently, there has been increasing interest in by-products as partial substitution of traditional feedstuffs in ruminant feeding. From a nutritional point of view, by-products are included in the ration to supply energy and protein, but are often also characterized by high fibre content. This is the case of orange pulp or soybean hulls, which are typically used as grain substitutes. A large number of the citrus by-products feedstuffs, including orange pulp, are suitable for inclusion in ruminant diets because of the ability of ruminants to ferment high fibre feeds in the rumen [2]. According to [3] the neutral detergent fibre (NDF) level of dry orange pulp is intermediate (25%) between barley grain (17%) and soy hulls (58%). Orange pulp contains relatively large amounts of pectin (25%) and sugars (23%) and low amounts of lignin (2%) and starch (0.5%), with a very limited amount of available nitrogen (6% of crude protein [CP]). Soybean hull has a similar CP content to that of barley grain (11%) and is high in NDF (58%, high in cellulose) but low in lignin (2%), NFC (24%) and sugars (1.5%), with no starch content (barley grain has 51% starch).

In ruminant nutrition, decreased production of methane (CH_4_) can represent an improvement in feed efficiency, as ruminants lose between 2–12% of their gross dietary energy in the form of CH_4_ [4]. Ruminant nutrition has been also demonstrated to be an efficient strategy to modify CH_4_ emissions from manure [5]. Moreover, [6] concluded that diet has the greatest effect on the quantity of CH_4_ produced from slurry.

Moreover, ruminants contribute to global warming through emission of nitrous oxide from urine and faeces. To reduce nitrogen (N) excretion and improve N efficiency in ruminants, dietary levels of N and optimal balance between N and energy substrates in the diet should be targeted.

Our hypothesis is that orange pulp and soybean hulls could replace cereal-based concentrate in goat diets without compromising energy and protein partitioning in lactating goats. The aim of this experiment was to study the effect of replacing barley grain in the mixed diet with dry orange pulp or soybean hulls on energy partitioning, enteric and manure derived CH_4_ emissions, carbon (C) and N balance and milk performance in dairy goats during midlactation.

## Materials and Methods

### Ethics statement

The experimental procedure was approved by the Animal Use and Care Committee of the Polytechnic University of Valencia (Spain) and followed the codes of practice for animals used in experimental works proposed by the [7]. Moreover, the Animal Science Department from the Polytechnic University of Valencia provided veterinary researchers who ensured that goat management followed the codes of practice for animals used in experimental works proposed by the European Union [7]. The authors declare that this manuscript does not infringe any ethical issues and involves no endangered or protected species.

### Animal and feeding

The experiment was conducted at the Animal Science Department Experimental Farm (ACUMA Research Centre), Valencia (Spain). Twelve multiparous mature Murciano-Granadina dairy goats in midlactation were selected and divided into three groups based on similar body weight (BW) and milk production (42.1 ± 1.2 kg and 2.16 ± 0.060 kg/goat/day, respectively). The experiment was conducted in an incomplete crossover design where one group of four goats was fed a mixed ration of barley grain, another group of four goats replaced barley grain with orange pulp and the last group of four goats did so with soybean hulls. Goats were fed above production level and ingredients and chemical composition of the three formulated diets are shown on Table 1. The Spanish ruminant production system [3] is based on high use of concentrate (40 to 70%), with mixed diets instead of whole forage rations. The total amount of feed offered was 2.4 kg per goat and day, as fed. Goats were fed mixed diets with 800 g of alfalfa hay per day and 1600 g of concentrate per goat and day (forage and concentrate ratio = 33/67). The concentrate was mixed and pelleted along with the premix. One group was fed concentrate with 670 g/kg DM of barley (BRL diet). The other two groups substituted barley grain with by-products: dry orange pulp (OP diet) and soybean hulls (SH diet). Requirements of the goats were obtained using the recommended values of [8,3]. Diets were supplemented with a salt vitamin-mineral premix and water was freely available at all times. Chemical composition shown in Table 1 is whole mixed ration (forage and pelleted concentrate). In order to achieve isoenergetic diets, bypass fat was added. The mean gross energy (GE) of the three diets was 18 MJ/kg DM. Mixed diets contained similar amounts of CP (13%, on DM basis). All goats were housed in a building in which the environment was partially controlled (HOBO; BoxCarPro3 software).

**Table 1:** Ingredients and chemical composition of the diets.

	Diet^1^		
Item	BRL	OP	SH
Ingredients, g/kg DM			
Alfalfa hay	330.0	330.0	330.0
Barley	594.2	-	-
Orange pulp	-	593.7	-
Soy hulls	-	-	595.3
Soy meal (44% CP)	46.8	59.3	39.6
Calcium carbonate	11.8	7.6	4.3
Sodium chloride	8.7	2.7	7.5
Bypass fat^2^	4.2	2.5	19.1
Premix^3^	4.2	4.2	4.3
Chemical composition, % of DM			
Dry matter	89.6	87.5	89.2
Organic matter	92.8	91.0	91.4
Crude Protein	13.7	11.9	14.0
Ether extract	2.2	1.1	4.8
Neutral detergent fibre	41.5	31.4	54.6
Acid detergent fibre	15.8	20.8	38.2
NFC^4^	35.5	46.6	21.0
Starch	32.7	5.1	1.8
Gross energy, MJ/kg DM^5^	17.9	16.9	18.0

^1^BRL = barley; OP = orange pulp; SH = soybean hulls.

^2^Bypass fat of palm fatty acid distillate. Provided by Norel Animal Nutrition, Norel S.A., Spain.

^3^Provided by NACOOP S.A., Spain. Premix composition (ppm or UI per kilogram of premix): Se, 40; I, 250; Co, 80; Cu, 3000; Fe, 6000; Zn, 23400; Mn, 29000; S, 60000; Mg, 60000; vitamin A, 2000000 UI; vitamin D3, 400000; vitamin E, 2000 ppm; nicotinic acid, 10000; choline, 20300.

^4^NFC = non fibrous carbohydrate content: 100-(NDF+ash+CP+EE).

^5^DM = dry matter.

### Experimental schedule and measurements

Apparent total tract digestibility, gas exchange, energy partitioning, C and N balance, oxidation of nutrients and milk performance were determined. Before moving the animals from group compartments to pens, the 12 goats selected were split into three groups of 4 goats for 15 days. Prior to each experimental period, the animals were adapted to the diet, environmental conditions of the metabolic cages and head hood by housing them individually for short periods of 20 minutes for 15 days. Then, goats were moved to individual pens for 7 days and during this time they were not moved periodically to the metabolic cages, feeding only on the experimental diets. The experiment was conducted as a crossover design with 12 lactating dairy goats kept in 3 groups and fed 3 dietary treatments in two periods; period one (7 days of adaptation to metabolic cages + 7 days of digestibility + 12 days of respirometry) and period two (10 days of diet adaptation + 5 days digestibility + 12 days respirometry). To this end, they were allocated to individual metabolism cages at thermoneutrality (20–23°C determined by a Hobo probe, ONSET data loggers, Cape Cod, MA, USA). The animal was not attached to the metabolic cage, as the lateral cage structure is adjustable, so goats could stand or lie down freely. Next, data on the feed offered and refused and the total faecal, urine and milk output were recorded daily for each goat over a 5-day period, as well as BW at the beginning and end of the period. Faeces were collected in wire-screen baskets placed under the floor of the metabolism crates and urine was collected through a funnel into plastic buckets containing 100 mL 10% (vol/vol) of H_2_SO_4_ to acidify the urine of each goat. The acidification of urine was necessary to prevent microbial degradation and the loss of volatile ammonia-N (NH_3_-N). Representative samples (10%) of diet, faeces and urine were collected over 5 consecutive days, stored at -20°C and pooled for chemical analysis. The goats were milked once daily at 0800 h with a portable milking machine (Flaco, model DL-170, J. Delgado S.A., Ciudad Real, Spain). Immediately after milking, the individual milk yield was measured and a sample of 10% was put in a bottle and frozen until analysis. In addition, samples were collected into a plastic vial containing 20 mg of potassium dichromate as a preservative and taken to the Interprofessional Dairy Laboratory of the Valencia Community Region (LICOVAL, Valencia, Spain) for compositional analysis (dry matter, crude protein, fat and lactose). At the end of the second digestibility trial, blood samples were collected from each goat. Jugular blood samples were taken before the morning feeding. Blood was sampled in 10 mL tubes treated with EDTA or Li-heparin, immediately centrifuged for plasma separation and stored at -20°C. Ruminal fluid samples were collected by stomach tube before the morning feeding on the last day of the apparent digestibility trial. Ruminal samples were taken only during the second period. Ruminal fluid pH was immediately determined using a Model 265A portable pH meter (Orion Research Inc., Beverly, MA, USA). A ruminal fluid sample was acidified with 50% H_2_SO_4_ and frozen until later determination of NH_3_-N. Samples for analysis of VFA were mixed with H_3_PO_4_ and kept frozen until analysis.

There is renewed interest in the measurement of energy metabolism based on open-circuit respirometry as an oblique consequence of the drive to reduce livestock greenhouse emissions. Open-circuit respirometry is an indirect calorimetry method that consists of measuring the gas exchange associated with the oxidation of energy substrates and determining the associated heat production. Thus, gas exchange was measured for each goat for 24 h in continuous by an indirect calorimetric system based on a ventilated head-box designed for small ruminants. To this end, 12 days were taken for each period (6 goats and 2 treatments), as we only have one indirect calorimetry unit. The respirometry system has a head hood, a flow meter (Thermal Mass Flowmeter Sensyflow VT-S, ABB, Alzenau, Germany) and air suction provided by a centrifugal fan (CST60 Soler Palau Inc., Parets del Vallès, Barcelona, Spain). The head hood was suspended on the front structure of the metabolic cage by two hooks placed on its rear side. The hood had a transparent acrylic window at the front and a drawer with a pulling handle to open and place the animal food and water in a bucket. The drawer was locked by two lateral locks situated on its front side and the main body of the head hood. A foam tape was placed on the edge of the drawer for an adequate seal. An opening (200 mm long × 520 mm high) in the rear panel of the hood was set up with a tightly woven nylon curtain (funnel shape) with a hole in the middle for the animal’s neck, fixed by four bolted platens and glued all around the opening edge. It was equipped with a nylon drawstring through a fold edge to fit and tie it around the neck to avoid gas leaking. During respirometry, the animal was attached to the front structure of the metabolic cage by a necklace and chain so that it could freely stand or lie down. Atmospheric air entered the head hood through an orifice (internal diameter 20 mm) made on its top on the opposite side of the main air suction line. The methane (CH_4_) and carbon dioxide (CO_2_) concentration were measured using the infrared principle and oxygen (O_2_) was measured by the paramagnetic principle (Easyflow Gas Analyzer, model 3020, ABB, Alzenau, Germany). Although the unit was an autocalibrated model, the analysers were calibrated with reference gases before each test. [9,10] described the mobile open-circuit respirometry system used for these measurements.

The whole system was calibrated by injecting pure N_2_ and CO_2_ into the head box [11], determined gravimetrically using a precision scale (MOBBA mini-SP 0.2–30 kg, Industrial Weighing System, Barcelona, Spain). Calibration factors were calculated according to [12]. The CH_4_ and CO_2_ production and O_2_ consumption were calculated as described by [13]. An initial atmospheric air sample was collected and the gas concentrations were used as reference for calculations.

### Ultimate CH_4_ yield (B_0_)

All faecal samples were pooled (500 g) by type of diet and stored at 4°C. The ultimate CH_4_ yield (B_0_) was assessed after incubating the faeces for a standard period of 90 days at 38°C. Five replicates were incubated for each treatment. Faeces (11 g) were introduced in 250 mL Pyrex glass bottles applying 1.6:1 sample to headspace ratio [14]. In parallel, bottles with faecal samples and sodium benzoate (17.5 mL) were incubated to check the likely inhibition of the methanogenesis [15]. Digested sewage sludge (17.5 mL) was also added as inoculum to all bottles. Distilled water and the inoculum were incubated as blanks. The bottles were capped with a thick rubber septum and the headspace was flushed with pure N_2_ to prevent O_2_ inhibition. Biogas production was monitored every 2–3 days by pressure measurement using a Delta Ohm manometer HD 9220 (absolute pressure meter 0–2,000 mbar ± 0.8%). Gas samples (5 mL) were collected from the headspace and inserted into a GC vial (9 mL). Samples were afterwards diluted with 10 mL N_2_. Biogas concentration was analysed by GC (GC-7890A, Agilent Technologies). The pH values of the different treatments were monitored throughout the experimental period. ISO standard methodology (11734:1999) was selected to estimate the B_0_. The net C mass (mh) and CH_4_ mass (mCH_4_) required by the ISO methodology were calculated after pressure measurements and CH_4_ analyses, respectively. The ultimate CH_4_ yield value was calculated as follows:

$$B_{0}=\left\{m_{CH4}\times \left(\frac{16,000}{12,000}\right)\times 0.67x10^{-6}\times 1000|Organic\;Matter\;incubated\right\}$$

### Chemical analysis

Feed, feed refusal and faeces samples were first dried in a forced air oven at 55°C for 48 h, then ground to pass a 1 mm screen before analysis. Urine and milk were dried by lyophilization. Chemical analyses of the diet, refusals and faeces were conducted according to [16] for DM, ash and ether extract (EE). The DM of diets and faeces was determined by oven-drying at 102 ± 2°C for 24 h. Ash concentration was measured by incineration in an electric muffle furnace at 550°C for 6 h to determine OM. The EE was extracted with petroleum ether after acid hydrolysis to recover saponified fat (Soxtec System HT Tecator, Hillerød, Denmark; 1047 Hydrolyzing Unit and 1043 Extraction Unit). The NDF and ADF were measured in an ANKOM Fiber Analyzer (A220, ANKOM Technologies, Fairport, NY, USA) according to [17,16], respectively. The NDF was determined using sodium sulphite and alpha amylase. Lignin was determined according to [18]. The NFC content of diets was calculated by difference method based on chemical analysis of individual feeds according to [19]: NFC = 100−NDF−ash−CP−EE. The GE content of the dried samples (feed, faeces, urine and milk) was analysed by combustion in an adiabatic bomb calorimeter (Gallenkamp Autobomb; Loughborough, UK). Starch content was determined by enzymatic method (α-amylase obtained from Sigma-Aldrich, Steinheim, Germany) according to [20]. The C and N were analysed by the Dumas principle (TruSpec CN; LECO Corporation, St. Joseph, MI, USA). Multiplying N by a factor of 6.25 converted the results to CP.

Milk composition (fat, protein, lactose, citrate and total milk solids content) was analysed with an infrared analyser (MilkoScan FT120 Foss Electric, Hillerød, Denmark). Fatty acid (FA) methyl esters of total milk lipids were prepared directly as previously described [21]. FA methyl esters were analysed in a Focus Gas Chromatograph (Thermo, Milan, Italy) equipped with a split/splitless injector and a flame ionization detector. Separation of methyl esters was performed in a fused silica capillary column SP^™^ 2560 (Supelco, PA, USA) (100 m x 0.25 mm x 0.2 μm film thickness). The carrier gas was Helium at a linear velocity of 20 cm/sec. The samples were injected with a split ratio of 1/100. The initial oven temperature was set at 140°C held for 5 min and increased to 240 at 4°C/min and finally maintained at that temperature for 30 min. Both detector and injector temperatures were set at 260°C.

A subset of milk samples was collected and analysed for milk urea. Urea and total protein were analysed in urine. In plasma glucose, non-esterified fatty acids (NEFA), ß-hydroxybutyrate (BHBA), ketone bodies and triglycerides were also analysed. All these samples were sent to a diagnostic lab (Laboratorio de Diagnóstico General, Comte Borrell, 08015 Barcelona, Spain) for determinations.

The NH_3_-N content of ruminal fluid samples and faeces were analysed by the Kjeldahl procedure (2300 Kjeltec Analyzer Unit Foss Tecator, Hillerød, Denmark). Determination of ruminal VFA was based on the method described by [22] using a gas chromatograph (Fisons 8000 series; Fisons Instruments SpA, Milan, Italy) equipped with a split/splitless injector and flame ionization detector.

### Calculations

The ME intake (MEI) was calculated as the difference between GE intake and energy losses in faeces, urine and CH_4_ (with an energy equivalent value of 39.5 kJ/L CH_4_) [23].

Quantitative measurements of gas exchange in respiration units have been used in indirect calorimetry to estimate the heat production in animals. Open-circuit respirometry is an indirect calorimetry method that consists of measuring the gas exchange associated with the oxidation of energy substrates and determining the associated heat production (HP). Brouwer [23] developed the equations for calculation of HP and net oxidation of protein, carbohydrate and fat in animals based on the respiratory quotient from measurements of gas exchange and nitrogen excretion in urine. The HP was determined from measurements of O_2_ consumption, CO_2_ and CH_4_ production, and urine N (N_urine_), using the equation of [23]:

$$HP(kJ)=16.18\times O_{2}+5.02\times CO_{2}-2.17\times CH_{4}-5.99\times N_{urine}$$

where gases were expressed in litres per day and N_urine_ in grams per day. The body tissue energy (TE_body_) was calculated as MEI—HP—milk energy (E_milk_). The energy associated with oxidation of protein (OXP), carbohydrate (OXCHO) and fat (OXF) was calculated by the method of [24,25] for ruminants. The CO_2_ production from oxidation (CO_2x_) was calculated as CO_2_−(CO_2_/CH_4_ × CH_4_), according to [26]. The calculations were carried out as follows:

$$OXP=6.25\times N_{urine}\times 18.42\left(kJ/g\right)$$

$$OXCHO=\left(-2.968\times O_{2}+4.174\times CO_{2x}-2.446\times N_{urine}\right)\times 17.58\left(kJ/g\right)$$

$$OXF=\left(1.719\times O_{2}-1.719\times CO_{2x}-1.963\times N_{urine}\right)\times 39.76\left(kJ/g\right)$$

Thus, the HP from oxidation (HPx) was:

$$HP_{x}(kJ)=16.18\times O_{2}+5.02\times CO_{2x}-5.99\times N_{urine}$$

Again, gases were expressed in litres per day and N_urine_ in grams per day. Heat of fermentation (HPf) was estimated subtracting HP from HPx. This method is termed the respiration quotient method (RQ) because it is based on determination of the respiratory quotient:

$$RQ=(litres\;CO_{2}|litres\;O_{2})$$

The non-protein respiratory quotient from oxidation of nutrients (RQnpx) was determined as:

$$RQnpx=(CO_{2x}-(N_{urine}\times 6.25\times 0.774))|(O_{2}-(N_{urine}\times 6.25\times 0.957))$$

Efficiency of use of ME for lactation was calculated according to [8]. Energy lost from the body, indicating mobilization of body fat reserves in support of milk secretion, was assumed to be used for milk synthesis with an efficiency of 0.84 and the concomitant energy storage during lactation was taken to be 0.95 times the milk secretion efficiency. Consequently, the corrected milk energy was estimated as E_milk_ + (0.84 x negative energy retention) + (1.05 x positive energy retention). The efficiency of use of ME for milk production (k_l_) was calculated as corrected milk energy divided by ME minus ME_m_), with MEm being the metabolizable energy for maintenance, which was obtained from [8] for goats (481 kJ/kg of BW^0.75^).

In the C and N balance, we followed the equation and values proposed by [11]. The C balance includes the measurement of carbon in feed and that voided in faeces, urine, CO_2_, CH_4_ and milk, while the N balance is based on the measurements on nitrogen in feed, faeces, urine and milk. The C balance gave the total amount of C retained in the body and the amount of C retained in fat was calculated by subtracting the amount of C retained in protein determined by the N balance. Assuming an energy equivalent of 39.76 kJ/g and a content of 0.767 C for fat, and 23.86 kJ/g and 0.16 N and 0.52 C for protein, the energy retained (kJ) in protein (TE_protein_) and fat (TE_fat_) was calculated, respectively, as:

$$TE_{protein}=N\;balance(g)\times 6.25\times 23.86$$

and

$$TE_{fat}=(C_{balance(g)}-N_{balance(g)}\times 6.25\times 0.52)\times 1.304\times 39.76$$

### Statistical analysis

The effects of starch on intake, digestibility, metabolic energy as well as C and N balance were analysed using the mixed model (proc MIXED) from [27]. The model for the dependent variables included the fixed effect of diet and period with goat as random effect. The following statistical model was used:

Y=μ+D+T+goat+DxT+ε

where Y is the dependent variable, μ is the overall mean, D and T are the fixed effects of diet and period of time, DxT their interaction, goat is the random effect of goat and ε is the random error. Least square means are reported throughout and differences were considered significant at P < 0.05.

## Results and Discussion

Most of the variables showed no significant effect for period of time in the crossover design, so the discussion was focused on the effect of diet.

### Feed Intake, digestibility and rumen fermentation

Dry matter intake (DMI) and total tract apparent digestibility of nutrients by Murciano-Granadina dairy goats are shown in Table 2. No significant effect on DMI was observed for diets, period of time and their interaction throughout the experiment (2.03 kg/d, on average). The effect of diet on digestibility was significant (P < 0.05) in DM, OM, CP, EE, ADF, starch and GE. The DM and GE total apparent tract digestibility were greater (P < 0.05) for the BRL diet (71.6% and 73.4% respectively, both DM basis) and the OP diet (73.5% and 73.4%, DM basis, respectively) than the SH diet (64% and 67.2%, DM basis, respectively). The higher NDF, ADF content and greater fat added in SH diet than BRL diet appeared to be the main factor responsible for the lower DM, OM and CP apparent digestibility found in SH diet. According to [19], soybean hull feed is a by-product that is highly digestible but low in non-fibrous carbohydrates. Difference (P < 0.05) was observed for the CP digestibility, being significantly lower for OP diet (56.4%, DM basis) than BRL diet (65.6%, DM basis), while SH diet (59.3%, DM basis) did not differ from the other two diets. Accordingly, diet OP has lower CP content and lower CP digestibility than the others, with a decrease in EE digestibility of 38 points compared to the BRL and SH diets. Decreased digestibility in CP and EE in diet OP affected rumen fermentation, as we observed in Table 3. Only GE digestibility scores were significantly higher in the second period than first one (73.1% and 70.3%, DM basis, respectively).

**Table 2:** Body weight, intake, and apparent digestibility coefficients of Murciano-Granadina goats (n = 12) during midlactation by type of diet and period.

	Diet^2^			Period^3^			*P-Value*		
Item^1^	BRL	OP	SH	1	2	SEM^4^	Diet	Period	Diet x Period
BW, kg	42.4	42.3	41.6	42.2	42.2	0.95	0.949	0.786	0.505
DMI, kg/d	2.0	2.0	2.1	2.0	2.0	0.02	0.248	0.259	0.244
Digestibility, % of DM									
DM	71.6^a^	73.5^a^	64.0^b^	70.3	69.7	0.93	0.001	0.579	0.871
OM	74.5^b^	77.5^a^	66.6^c^	73.4	72.9	1.00	0.001	0.554	0.927
CP	65.6^a^	56.4^b^	59.3^a^^b^	60.9	61.8	1.29	0.006	0.605	0.717
EE	74.2^a^	34.0^b^	71.1^a^	60.0	64.8	4.36	0.000	0.365	0.568
NDF	64.0	61.4	60.8	63.6	61.2	0.75	0.145	0.117	0.509
ADF	47.9^c^	65.6^a^	58.7^b^	55.6	55.7	1.78	0.001	0.964	0.920
Starch	99.4^a^	98.2^b^	91.9^c^	97.0	97.0	0.70	0.001	0.886	0.041
GE	73.4^a^	73.4^a^	67.2^b^	70.3	73.1	0.89	0.001	0.021	0.238

^a,b,c^Means within a row with different superscripts differ (P < 0.05).

^1^BW = body weight; BW^0.75^ = metabolic body weight; DMI = dry matter intake; DM = dry matter; OM = organic matter; CP = crude protein; EE = ether extract; NDF = neutral detergent fibre; ADF = acid detergent fibre; GE = gross energy.

^2^BRL = barley; OP = orange pulp; SH = soybean hulls.

^3^1 = first period; 2 = second period.

^4^ SEM = standard error of the mean.

**Table 3:** pH, ammonia-N (NH_3_-N), and VFA of Murciano-Granadina goats (n = 12) during midlactation by diet type.

	Diet^2^				*P-Value*
Item^1^	BRL	OP	SH	SEM^3^	Diet
pH	7.30^a^	7.10^b^	7.10^b^	0.041	0.031
NH_3_-N, mg/dL	15.08^b^	52.30^a^	38.33^a^	7.405	0.039
Total VFA, mM	14.16^c^	25.97^a^	23.94^b^	3.289	0.016
Individual VFA, mol/100 mol					
Acetic acid, C2:0	62.22^a^	56.98^b^	61.48^a^	1.435	0.031
Propionic acid, C3:0	13.42	14.98	15.90	0.624	0.293
Isobutyric acid, C4:0iso	3.86	2.87	3.19	0.362	0.591
Butyric acid, C4:0	14.39^b^	19.54^a^	13.09^b^	1.389	0.023
Isovaleric acid, C5:0iso	4.34	3.81	3.74	0.422	0.859
N-Valeric acid, C5:0	1.78	1.70	1.74	0.147	0.980
N-Caproic acid, C6:0	0.00^b^	0.12^b^	0.62^a^	0.103	0.004
Heptanoic acid, C7:0	0.00^b^	0.00^b^	0.23^a^	0.056	0.004

^a,b,c^Means within a row with different superscripts differ (P < 0.05).

^1^NH_3_-N = ammonia nitrogen; C = carbon; N = nitrogen.

^2^BRL = barley; OP = orange pulp; SH = soybean hulls.

^3^SEM = standard error of the mean.

Rumen fermentation parameters obtained are displayed in Table 3. It was not possible to assess the effect of period and interaction with diet because rumen liquid extraction was done only during the second period of the trial. Lower pH values were found in OP and SH than BRL (7.1 *vs*. 7.3, respectively). The greater values of pH found were probably associated with the contact of rumen liquid with saliva when the oesophageal tube was removed during sampling. An increase (P < 0.05) in acetic acid (C2:0) in the rumen when goats were fed the higher NDF diets was observed (62.22 and 61.48 *vs*. 56.98 mol/100 mol for diet BRL, SH and OP, respectively). Butyric acid (C4:0) was higher (P < 0.05) in OP diet compared to other diets and the highest numerical value of NH_3_-N was found in OP diet (52.3 mg/dL). The N-caproic acid (C6:0) and heptanoic acid (C7:0) showed significant (P < 0.05) differences between SH diet and OP or BRL diet. Goats fed fibrous by-products (OP and SH diets) had greater NH_3_-N than BRL (45.32 mg/dL for OP and SH on average *vs*. 15.08 mg/dL for BRL diet). Therefore, in our study the diet has the same source of protein (soybean meal) and different types of carbohydrate (starch, digestible and indigestible fibre), so the greater NH_3_-N found in fibrous diets (OP and SH) seems indicative of their inefficient use for ruminal proteosynthesis [28]; OP diet has a lower level of CP (12% on DM basis) than BRL and SH (14%, on DM basis and average), and SH the greatest content of EE (4.8% *vs*. 1.8% on DM basis for SH and others, respectively).

### Energy balance

The average values obtained for the calibration factor for the indirect calorimetry system, by releasing a known volume of N_2_ or CO_2_ into the respirometry system, were 1.0067 ± 0.00119 and 0.9979 ± 0.00823 for O_2_ and CO_2_, respectively.

Daily energy balance obtained with the three diets is shown in Table 4. Period of time only affected HP (being greater during the second period) and body energy retention (being negative in the first period and positive in the second period). These differences were in agreement with the physiology of dairy animals [29], where reserves mobilization took place close to peak lactation and was subsequently recovered.

**Table 4:** Daily energy partitioning (kJ/kg of BW^0.75^) of Murciano-Granadina goats (n = 12) during midlactation by type of diet and period.

	Diet^2^			Period^3^			*P-Value*		
Item^1^	BRL	OP	SH	1	2	SEM^4^	Diet	Period	Diet x Period
GEI	2170.2	2055.8	2296.2	2142.5	2205.6	42.66	0.113	0.451	0.243
E_feces_	582.0^b^	545.0^b^	758.9^a^	651.6	605.8	26.06	0.001	0.251	0.416
E_urine_	40.4^b^	56.7^a^	69.0^a^	50.3	60.4	5.24	0.041	0.344	0.916
E_methane_	135.9	141.5	135.4	129.7	145.5	4.60	0.852	0.111	0.231
MEI	1411.7	1312.5	1333.0	1310.9	1393.9	29.51	0.301	0.167	0.177
HP	817.6	847.1	803.8	795.2^b^	848.6^a^	14.06	0.471	0.033	0.312
E_milk_	513.5	474.6	527.8	572.0	492.3	14.65	0.473	0.052	0.027
TE_body_	80.5^a^	-9.2^b^	1.3^b^	-5.1^b^	74.1^a^	26.06	0.017	0.021	0.981

^a,b^ Means within a row with different superscripts differ (P < 0.05).

^1^GEI = gross energy intake; E_feces_ = energy losses in faeces; E_urine_ = energy losses in urine; E_methane_ = energy losses in methane; MEI = metabolizable energy intake; HP = heat production; RE_total_ = total recovered energy; RE_milk_ = recovered energy in milk; RE_body_ = recovered energy in tissue (TEbody = MEI − HP − Emilk).

^2^BRL = barley; OP = orange pulp; SH = soybean hulls.

^3^1 = first period; 2 = second period.

^4^ SEM = standard error of the mean.

Regarding the effect of the diet, no significant differences were observed for GE intake (2174.1 kJ/ kg of BW^0.75^, on average) and significantly higher (P < 0.05) energy losses in faeces (E_feces_) were found for SH diet than the BRL or OP diets, possibly due to the higher level of fat in the SH diet (4.8 *vs*. 1.7% for SH and others, respectively). The OP and SH diets presented similar energy losses in urine (62.9 kJ/kg of BW^0.75^, on average) and were significantly higher (P < 0.001) than BRL (40.4 kJ/kg of BW^0.75^), indicating that increasing the amount of NH_3_-N in rumen liquid augmented the energy losses in urine. The CH_4_ energy losses were not different among treatments. No differences were observed in MEI, with average values of 1352.4 kJ/ kg of BW^0.75^. A significant effect of period of time (P < 0.05) was observed in HP, with the highest values for the second one (848.6 kJ/ kg of BW^0.75^ *vs*. 795.2 kJ/ kg of BW^0.75^, respectively). No statistically significant differences were observed in E_milk_. The TE_body_ was significantly affected (P < 0.05) for the diet and period of time. Positive energy balance (80.5 kJ/ kg of BW^0.75^) was found for the starchy diet (BRL), around zero (1.3 kJ/ kg of BW^0.75^) in diet greater in NDF (SH) and negative energy balance (-9.2 kJ/ kg of BW^0.75^) in diet rich in digestible fibre (OP). Analysing the period of time, almost zero energy balance (-5.1 kJ/ kg of BW^0.75^) was found in the first period and positive balance (74.1 kJ/ kg of BW^0.75^) in the second period (P < 0.05).

The efficiency of use of ME for milk production (k_l_), according to [8], was calculated as E_milk_ output adjusted to zero energy balance divided by ME-MEm, and MEm was obtained from the estimation according to [8] for qm of 0.62 (481 kJ/kg of BW^0.75^). No significant differences were observed between diets for k_l_ (0.61 on average). This value was analogous to those obtained by other authors [29] with lactating Granadina goats (0.67), and [30] with Alpine goats during midlactation and 60% of concentrate (0.63). According to [31], a value for mixed diet in mid lactation of 0.67 for Saanen goats was found, and [32], with mixed diets and goats at mid lactation and positive energy balance, reported values around 0.63.

### Oxidation of nutrients

CO_2_ production is derived from nutrient oxidation and rumen fermentation. The separation between these two components is necessary to calculate the substrate oxidation in ruminants and determine the proportion of substrate oxidation supporting the total HP associated with oxidative processes. The proportional contribution to HPx due to oxidation of nutrients is shown in Table 5. No effect of interaction between diet and period was observed. Diet had no significant effect on HPx (789 kJ/kg of BW^0.75^, on average). The significant (P < 0.05) effect of period of time was higher during the second period than the first one for HPx and OXP. A significant effect of diet (P < 0.05) was observed in HPf and OXF, being higher for SH than the other two diets. The more fibrous diets (higher NDF in SH diet) were accompanied by greater HPf than starch (BRL) or digestible fibre (OP) diets. The OXCHO was higher in BRL diet than others, possibly associated with the greater amount of starch in the BRL diet than in OP and SH.

**Table 5:** Heat production (kJ/kg of BW^0.75^) from oxidation and fermentation; daily oxidation (kJ/kg of BW^0.75^) of protein, carbohydrate, and fat and their contribution to the heat production from oxidation substrates (%) of Murciano-Granadina goats (n = 12) during midlactation by type of diet and period.

	Diet^2^			Period^3^			*P-Value*		
Item^1^	BRL	OP	SH	1	2	SEM^4^	Diet	Period	Diet x Period
HPx	795.7	811.7	759.7	765.6	815.0	13.47	0.458	0.033	0.329
HPf	21.9^c^	35.4^b^	44.1^a^	29.7	33.6	2.32	0.000	0.268	0.442
OXP	56.0	47.5	66.4	49.1^b^	63.9^a^	3.45	0.083	0.014	0.489
OXCHO	471.9^a^	363.0^b^	115.6^c^	329.0	361.0	35.65	0.001	0.123	0.219
OXF	267.7^c^	401.1^b^	577.5^a^	387.3	389.9	33.23	0.001	0.505	0.148
OXP/HPx	7.1	5.8	8.8	6.4	7.9	0.44	0.056	0.054	0.733
OXCHO/HPx	59.2^a^	45.0^b^	14.6^c^	42.1	44.3	4.28	0.001	0.266	0.178
OXF/HPx	33.7^c^	49.2^b^	76.6^a^	51.5	47.8	4.40	0.001	0.175	0.186
RQnpx	0.89^a^	0.84^b^	0.76^c^	0.82	0.84	0.01	0.001	0.191	0.142

^a,b,c^Means within a row with different superscripts differ (P < 0.05).

^1^HPx = heat production from oxidation of nutrients; HPf = heat production of fermentation [HPf = HP − HPx (Brouwer, 1958)]; OXP = heat production associated with the oxidation of protein; OXCHO = heat production associated with the oxidation of carbohydrates; OXF = heat production associated with the oxidation of fat; RQnpx = non-protein respiratory quotient (unitless) from oxidation of nutrients {[CO_2_x − (Nurine× 6.25 × 0.774)]/[O_2_ − (Nurine × 6.25 × 0.957)], where CO_2_ = CO_2_ production from oxidation and Nurine = N in urine}.

^2^BRL = barley; OP = orange pulp; SH = soybean hulls.

^3^1 = first period; 2 = second period.

^4^SEM = standard error of the mean.

Therefore, the heat from OXP contributed approximately 7.2% of HPx on average for three treatments. The BRL diet oxidized 59.2% of nutrients as OXCHO and only 33.7% as OXF. However, nutrient oxidation as OXF increased significantly (P < 0.05) to 76.6% in SH, and nutrient oxidation as OXCHO decreased significantly (P < 0.05) to 14.6%. The higher amount of NFC in OP diet promotes the higher OXCHO compared to SH diet. Nevertheless, OP diet presented intermediate values compared to the other two diets; OXCHO and OXF were 45% and 49.2%, respectively. Few studies relating to oxidation of nutrients are available for ruminants. In the study of [32] in goats during midlactation, the corn grain in the total mixed diets was replaced with a blend of fibrous by-product such as soy hulls and corn gluten feed. In that trial, no differences were observed between diets for OXCHO and OXF (55% and 29% on average, respectively). Hence, similar OXCHO to that in our study was obtained, but OXF was lower than in our trial. So, we expect similar OXF in our SH diet to that in the blend of fibrous by-products from [32]. These latter authors added almost 4% of fat to the fibrous diet, which may have affected fibre fermentation [33]. Other studies in calves [25] with positive retained energy as fat pointed out that part of the OXF should originate from ingested carbohydrate, mainly fibre. Significant difference (P < 0.05) was observed for RQnpx, being significantly lower for SH (0.76) than OP (0.84), both of which were lower than BRL (0.89). As stated by [25], RQnpx lower than 1 indicates predominance of OXF *vs*. OXCHO, as we found in our study with the OP and SH diets.

### Carbon and nitrogen balance

The daily C and N balance and the calculated tissue recovered as protein and fat are displayed in Table 6. No effect of interaction between diet and period was observed, with the exception of C in milk. Although the interaction was significant, the effect of diet or period of time was not significant for C in milk and the effect of diet on milk chemical composition was not significant (Table 7). More significant differences were found for the excretion of C in faeces in SH diet (19.8 g/ kg of BW^0.75^) than in the others (14.35 g/ kg of BW^0.75^ on average), associated with the greater content in fat that was not absorbed, as mentioned above. The C in CO_2_ expired was also statistically different for both time periods: 22.5 g/ kg of BW^0.75^ in the second period of the trial and 20.8 g/ kg of BW^0.75^ during the first. The C secreted into the milk was not significantly affected by treatment. The C retained was significantly affected (P < 0.05) for the effect of diet and period of time.

**Table 6:** Carbon and nitrogen balance (g/kg of BW^0.75^) of Murciano-Granadina goats (n = 12) during midlactation by type of diet and period.

	Diet^2^			Period^3^			*P-Value*		
Item^1^	BRL	OP	SH	1	2	SEM^4^	Diet	Period	Diet x Period
C_intake_	52.4	50.1	56.1	52.0	53.2	1.04	0.103	0.446	0.242
C_feces_	15.0^b^	13.7^b^	19.8^a^	15.7	16.3	0.70	0.001	0.369	0.555
C_urine_	1.3^b^	1.6^b^	1.8^b^	1.5	1.5	0.09	0.045	0.728	0.427
C_CO2_	21.5	22.6	21.1	20.8^b^	22.5^a^	0.44	0.366	0.026	0.244
C_CH4_	1.8	1.9	1.8	1.8	1.9	0.08	0.851	0.111	0.231
C_milk_	11.1	10.5	11.4	11.6	10.4	0.30	0.408	0.089	0.027
C_retained body_	1.8^a^	0.2^b^	-0.2^c^	1.1^a^	0.5^b^	0.51	0.026	0.045	0.701
N_intake_	2.7	2.3	2.9	2.6	2.6	0.06	0.003	0.460	0.238
N_feces_	0.9	1.0	1.2	1.0	1.0	0.04	0.075	0.978	0.870
N_urine_	0.5^b^	0.4^b^	0.6^a^	0.4^b^	0.6^a^	0.03	0.043	0.014	0.498
N_milk_	0.8	0.7	0.8	0.8	0.8	0.02	0.044	0.248	0.075
N_retained body_	0.4^a^	0.2^b^	0.3^a^	0.4	0.3	0.04	0.043	0.145	0.196
TE _protein_, kJ/kg of BW^0.75^	64.0^a^	28.3^c^	50.0^b^	58.5	42.2	11.52	0.023	0.238	0.552
TE _fat_, kJ/kg of BW^0.75^	19.2^a^	-40.6^b^	-45.0^b^	-9.3^b^	-20.0^a^	10.56	0.045	0.048	0.506

^a,b,c^Means within a row with different superscripts differ (P < 0.05).

^1^C_intake_ = C intake; C_feces_ = C losses in faeces; C_urine_ = C losses in urine; C_CO2_ = C losses in CO_2_; C_CH4_ = C losses in methane; C_milk_ = recovered C in milk; C_retained body_ = recovered C in tissue; N_intake_ = N intake; N_feces_ = N losses in faeces; N_urine_ = N losses in urine; N_milk_ = recovered N in milk; N_retained body_ = recovered N in tissue.

^2^BRL = barley; OP = orange pulp; SH = soybean hulls.

^3^1 = first period; 2 = second period.

^4^SEM = standard error of the mean.

**Table 7:** Daily milk production and composition of Murciano-Granadina goats (n = 12) during midlactation by type of diet and period.

	Diet^1^			Period^2^			*P-Value*		
Item	BRL	OP	SH	1	2	SEM^3^	Diet	Period	Diet x Period
Milk yield, kg/goat/day	2.29	2.01	2.17	2.38^a^	1.93^b^	0.081	0.247	0.004	0.612
Composition, %									
DM^4^	14.7	15.7	15.4	14.5^b^	16.0^a^	0.28	0.296	0.010	0.553
Fat	5.4	6.3	6.4	5.7	6.3	0.21	0.073	0.121	0.602
Protein	4.0	3.7	3.7	3.3^b^	4.2^a^	0.13	0.544	0.001	0.226
Lactose	4.6	4.7	4.5	4.6	4.6	0.05	0.613	0.906	0.220

^a,b^Means within a row with different superscripts differ (P < 0.05).

^1^BRL = barley; OP = orange pulp; SH = soybean hulls.

^2^1 = first period; 2 = second period.

^3^SEM = standard error of the mean.

^4^DM = dry matter.

Goats in all three groups ingested similar amounts of N (2.63 g/kg of BW^0.75^, on average) and no differences were found in excreted N (1.03 g/kg of BW^0.75^, on average). The N losses in urine were greater for SH treatment (0.6 g/kg of BW^0.75^) when compared to BRL and OP (0.45 g/kg of BW^0.75^, on average). The N secreted into the milk was not affected by treatment (0.77 g/kg of BW^0.75^, on average). The N balance was different (P < 0.05) between OP and the other two diets. The N balance was positive for all treatments. Although some authors [34] indicate reduction in urinary N output when ME intake increase, in our study we did not find any differences.

The values of C and N retained in the body were converted to tissue energy recovered as protein or fat, and differences (P < 0.05) were found. The TE_protein_ was 64, 28.3 and 50 kJ/kg of BW^0.75^ for BRL, OP and SH, respectively. Regarding TE_fat_, the OP and SH diets showed greater (P < 0.05) fat mobilization (-42.82 kJ/kg of BW^0.75^, on average) than BRL (19.2 kJ/kg of BW^0.75^), as mentioned above (Table 4).

### Milk production and fatty acids

Table 7 reports milk yield and chemical composition of the goats during the experiment. Significant (P < 0.05) and higher milk yield were found for the first period (2.38 *vs*. 1.93 kg/d for first and second period, respectively). Milk dry matter and protein content were statistically different (P < 0.05) between the two periods, with greater values for the second period than for the first (14.5 *vs*. 16% for dry matter and 3.3 and 4.2% for protein content). The first period of time is closer to post-partum, which is when lower milk chemical composition and higher milk production are observed in dairy ruminants [30]. No effect of interaction between diet and period was observed. Diet had no effect on milk yield (average milk yield was 2.16 kg/d), dry matter, fat, protein and lactose (15.25%, 6.0%, 3.75% and 4.6% on average, respectively). Although increased milk fat content is common when dietary fibre concentrations rise at the expense of starch [35], no differences were found in our study.

Effect of diet on the fatty acid profile of milk fat is shown in Table 8. No effect of diet was observed in fatty acids with 4 to 15 carbon atoms. However, there were significant differences between diets in most of the other fatty acids. The fatty acids with 16 or fewer carbon atoms derive from *de novo* synthesis, whereas those with 18 or more carbon atoms come from the diet or from lipid mobilization [35]. Significant differences (P < 0.05) were observed for palmitic acid (C16:0) and cis-10-heptadecenoic acid (C17:1), being significantly higher for OP diet than BRL diet, and SH diet did not differ from the other two diets. Pentadecanoic acid (C15:0) and heptadecanoic acid (C17:0) are potential biomarkers of rumen function, as they are found in rumen bacterial lipids and might be partially synthesized endogenously from rumen substrates in the mammary gland [36,37,38]. The differences (P < 0.05) found between treatments (lower content of pentadecanoic acid (C15:0) in the milk of OP and SH goats than BRL) suggest a negative impact of these diets on rumen bacterial metabolism and fermentative activity. Our ammonia-N results found in rumen liquid are in accordance with the differences observed in these fatty acids. Moreover, a greater amount (P < 0.05) of heptadecanoic acid (C17:0) in milk fat from OP and SH diets probably reflects a larger contribution of mobilized fat [37], as we observed in Table 6; -42.82 kJ TEfat/kg of BW^0.75^ in OP and SH diet on average against 19.2 kJ TEfat/kg of BW^0.75^ in BRL. Authors [36] suggest that the amount of starch in the diet is an important factor determining high milk elaidic acid (C18:1n9t) content (greater in BRL than others). Milk fat of goats fed BRL diet had lower percentages of vaccenic acid (C18:1n7) and higher of linoleic acid (C18:2n6c) and cis-11-eicosenoic acid (C20:1) than the other two diets. Higher vaccenic acids (C18:1n7) in OP and SH diets seems to be positively correlated with negative energy balances in goats. Another study [39] found a positive correlation between negative energy balance and oleic acid (C18:1n9c) in dairy cows. Goats fed OP diet had greater percentages of linolenic acid and CLA 10t12c in their milk fat than goats fed BRL or SH diet. Significant differences were observed for CLA 9c11t + 9t11c and arachidonic acid, being significantly higher for BRL diet than SH diet, and OP diet did not differ from the other two diets. Milk from goats fed BRL diet is the richest in medium-chain fatty acids and polyunsaturated fatty acids. Milk from goats fed SH diet is the richest in monounsaturated fatty acids and goats fed OP diet have milk with more saturated fatty acids. Atherogenicity index was calculated as indicated by [40]. The milk of goats fed SH diet has a lower atherogenicity index than others (4.42 *vs*. 5.4 on average, respectively), but differences were not significant.

**Table 8:** Fatty acid composition (g/100 g of identified fatty acids) of milk fat for goats (n = 12) fed the experimental diets.

	Diet^2^				*P-Value*
Item^1^	BRL	OP	SH	SEM^3^	
Butyric Acid, C4:0	0.30	0.32	0.31	0.007	0.429
Caproic Acid, C6:0	0.95	0.97	0.92	0.025	0.760
Caprylic Acid, C8:0	1.82	1.74	1.68	0.050	0.539
Capric Acid, C10:0	10.03	9.60	8.81	0.236	0.098
Undecanoic Acid, C11:0	0.37	0.32	0.29	0.022	0.321
Lauric Acid, C12:0	6.89	5.99	5.46	0.297	0.119
Myristic Acid, C14:0	13.38	13.13	11.94	0.327	0.185
Myristoleic Acid, C14:1	0.28	0.30	0.27	0.025	0.878
Pentadecanoic Acid, C15:0	0.22^a^	0.14^b^	0.15^b^	0.002	0.247
Palmitic Acid, C16:0	40.61^b^	44.53^a^	42.63^a^^b^	0.620	0.021
Palmitoleic Acid, C16:1	1.11	1.36	1.27	0.074	0.391
Heptadecanoic Acid, C17:0	0.59^b^	0.70^a^	0.71^a^	0.023	0.039
cis-10-Heptadecenoic Acid, C17:1	0.28^b^	0.36^a^	0.33^a^^b^	0.015	0.047
Stearic Acid, C18:0	4.69	3.77	4.96	0.324	0.375
Elaidic Acid, C18:1n9t	0.37^a^	0.32^a^	0.23^b^	0.003	0.019
Oleic Acid, C18:1n9c	13.43^b^	12.45^b^	16.70^a^	0.616	0.017
Vaccenic Acid, C18:1n7	0.31^b^	0.46^a^	0.41^a^	0.018	0.001
Linoleic Acid, C18:2n6c	3.18^a^	2.25^b^	1.95^b^	0.150	0.001
Arachidic Acid, C20:0	0.11^b^	0.10^b^	0.16^a^	0.006	0.001
gamma-Linolenic Acid, C18:3n6	0.00	0.00	0.00	0.002	0.572
cis-11-Eicosenoic Acid, C20:1	0.07^a^	0.03^c^	0.05^b^	0.004	0.000
Linolenic Acid, C18:3n3	0.35^b^	0.52^a^	0.34^b^	0.027	0.008
CLA 9c11t + 9t11c	0.47^a^	0.41^a^^b^	0.28^b^	0.027	0.006
CLA 10t12c	0.01^c^	0.04^a^	0.02^b^	0.003	0.001
Arachidonic Acid, C20:4n6	0.19^a^	0.16^a^^b^	0.13^b^	0.010	0.036
Medium-chain fatty acids	20.04^a^	18.62^a^^b^	17.16^b^	0.505	0.051
Monounsaturated fatty acids	15.84^b^	15.30^b^	19.25^a^	0.603	0.013
Polyunsaturated fatty acids	4.22^a^	3.38^b^	2.72^b^	0.187	0.001
Saturated fatty acids	79.94	81.32	78.02	0.586	0.106
AI	5.15	5.64	4.42	0.218	0.076

^a-c^Means within a row with different superscripts differ (P < 0.05).

^1^CLA = conjugated linoleic acid; AI = Atherogenicity index calculated as C12:0 + 4 × C14:0 + C16:0/unsaturated fatty acids (Ulbricht and Southgate, 1991).

^2^BRL = barley; OP = orange pulp; SH = soybean hulls.

^3^SEM = standard error of the mean.

### Methane emission

Table 9 shows enteric CH_4_ emissions from goats. The average CH_4_ emission from the goats’ digestive tracts (enteric fermentation) was similar between diets and averaged at 57.4 L/goat per day. Methane conversion ratio, also designated Ym factor, represents energy loss as CH_4_ per unit of GE intake. No differences were observed and the average Ym value was 6.4. According to [4,41], fermentation of fibrous carbohydrates produces more CH_4_ than fermentation of soluble sugars, which in turn produce more CH_4_ than fermentation of starch. The similar CH_4_ production that we found in this work can be explained by the fact that the greater NH_3_-N found in OP diet probably cause asynchrony in rumen fermentation. Regarding the SH diet, the higher fat added (almost 5% *vs*. 1.7% for SH and others, respectively) probably affected fibre degradation, and this would be the reason for the greater NH_3_-N also found. Increasing the lipid content of the diet is acknowledged as a CH_4_ mitigation strategy due to reduction of methanogens [41], and the fat added probably reduced the CH_4_ production expected in SH diet. Significantly (P < 0.05) higher g CH_4_/kg milk was observed in the second period of the trail. No effect of interaction between diet and period was observed. No effects of CH_4_ emission were found when it was related to DM or OM.

**Table 9:** Methane emission of Murciano-Granadina goats (n = 12) during midlactation by type of diet and period.

	Diet^2^			Period^3^			*P-Value*		
Item^1^	BRL	OP	SH	1	2	SEM^4^	Diet	Period	Diet x Period
CH_4_, L/d	57.0	59.6	55.5	54.6	60.0	1.98	0.761	0.166	0.608
Ym, %	6.3	6.9	5.9	6.1	6.6	0.21	0.170	0.187	0.732
CH_4_/DMi, g/kg	20.1	21.1	19.0	19.3	20.9	0.61	0.498	0.183	0.717
CH_4_/OMi, g/kg	21.7	23.1	20.7	20.9	22.7	0.67	0.467	0.177	0.715
CH_4_/milk, g/kg	18.3	21.4	18.4	16.2^b^	22.1^a^	0.91	0.131	0.001	0.928

^a,b^Means within a row with different superscripts differ (P < 0.05).

^1^Ym = methane energy/gross energy intake; DMi = dry matter intake; OMi = organic matter intake.

^2^BRL = barley; OP = orange pulp; SH = soybean hulls.

^3^1 = first period; 2 = second period.

^4^SEM = standard error of the mean.

Table 10 shows the chemical composition of faecal samples incubated for B_0_ determination. The use of different carbohydrate sources in BRL, OP and SH diets significantly affected the OM, CP and NDF contents of the corresponding faeces. Faeces derived from BRL and SH diets showed higher OM and fibre content than OP faeces (P < 0.05). However, the fibre quality changed between BRL and SH faeces, as cellulose to hemicellulose ratio was different (P < 0.05). The higher cellulose content of SH faeces was in accordance with the high cellulose concentration of SH diet. From the different compounds of faeces, [42] concluded that lipids and proteins had the highest CH_4_ potential, while cellulose and lignin had the lowest. Moreover, [43] also showed that faecal CH_4_ production was limited when low fermentable fibre was found in faeces. The methanogenic potential of BRL, OP and SH faeces tended to support these previous conclusions as B_0_ values ranked according to their cellulose-lignin contents. Barley treatment averaged 109 L CH_4_ kg/OM, whereas mean B_0_ for SH and CP treatments were 102 and 113 L CH_4_ kg/OM, respectively. Nonetheless, B_0_ values were not significantly different among BRL, OP and SH diet-derived faeces. Taking into account the lignocellulose contents of the different treatments (Table 1), a further difference would have been expected in B_0_ values, especially between SH and OP faeces. However, the relatively high ash and lignin contents of OP faeces could have limited its methanogenic potential. The heating process associated with the drying of orange pulps could have caused that the lignin present in OP faeces had reacted with other molecules, resulting in products difficult to degrade [44]. In this sense, lignin concentration in OM has been identified as the strongest predictor of B_0_ [45].

**Table 10:** Mean faeces composition (%) before incubation for B_0_ determination by type of diet and period.

	Diet^2^			Period^3^			*P-Value*		
Item^1^	BRL	OP	SH	1	2	SEM^4^	Diet	Period	Diet x Period
DM	37.7^a^	38.4^a^	32.8^b^	36.7	33.5	0.83	0.047	0.052	0.419
Ash	16.6^b^	24.6^a^	15.9^c^	18.2	18.5	0.73	0.001	0.805	0.386
OM	83.5^a^	78.7^b^	85.0^a^	81.8	81.5	0.73	0.001	0.805	0.386
CP	17.1^a^^b^	18.6^a^	16.0^b^	17.7	16.9	0.44	0.004	0.312	0.766
NDF	53.2^a^	36.0^b^	52.1^a^	52.1	53.9	1.31	0.001	0.072	0.251
Cellulose	22.5^b^	16.9^c^	33.0^a^	18.1^b^	20.7^a^	1.22	0.001	0.022	0.319
Hemicellulose	22.2^a^	10.3^b^	11.5^b^	16.3	15.7	1.62	0.001	0.279	0.918
Lignin	8.5^a^	8.8^a^	7.6^b^	7.8	7.7	0.21	0.001	0.826	0.734

^a,b,c^Means within a row with different superscripts differ (P < 0.05).

^1^DM = dry matter; OM = organic matter; CP = crude protein; NDF = neutral detergent fibre; ADF = acid detergent fibre.

^2^BRL = barley; OP = orange pulp; SH = soybean hulls.

^3^1 = first period; 2 = second period.

^4^SEM = standard error of the mean.

**Fig 1:**
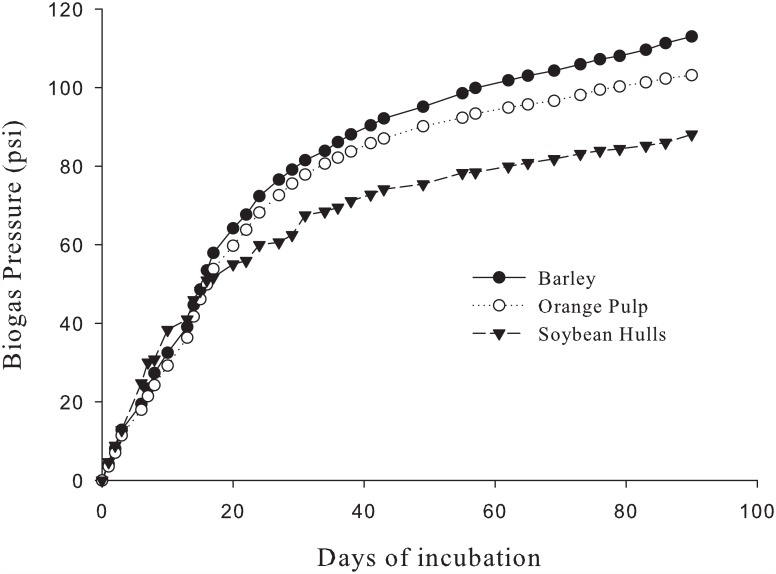
Organic matter degradation profile expressed as pressure (biogas) production.

All B_0_ values were lower than the default value reported by [46] for goats (180 L CH_4_ kg/OM). The lower B_0_ values allocated to BRL, OP and SH treatments were not accounted for by either the inhibition of OM degradation or the methanogenic process. In accordance with [15], OM degradation of all treatments was above 60% compared to faecal samples incubated with sodium benzoate. The OM degradation profile, expressed as pressure (biogas) production in Fig 1, was not different among the treatments within the first 20 days of incubation. Thereafter, OM degradability tended to decrease according to the lignocellulose contents of incubated faeces. The pH values and free NH_3_-N contents of BRL, OP and SH samples did not reach inhibitory values for methanogenesis throughout the experimental period. The pH ranged between 7.0 and 7.5 and free ammonia content was below 100 mg NH_3_-N/L [14]. Methane accounted for 60% (±6.3%) of biogas produced in all treatments, similar to values reported by [47].

### Metabolites in milk, urine and plasma

Table 11 shows metabolites from goats. It was not possible to assess the effect of period and interaction with diet, as blood samples were only taken during the second period of the experiment. The lower NH_3_-N found in diet BRL was followed by higher values (P < 0.05) of urea in urine, plasma urea and milk urea than the values found in OP diet, while SH diet was similar to BRL. The review of [48] reported the effect of the type of carbohydrate on rumen NH_3_ utilization and observed that starch and glucose rich diets resulted in lower concentration of rumen ammonia, plasma urea nitrogen and milk urea nitrogen. In our study the main source of protein was soybean meal for the three diets, although the (carbohydrate) energy sources for microbial protein synthesis were different. So, the type of carbohydrate affected rumen microbial protein synthesis because differences in NH_3_-N and fat mobilization were found. The BRL diet showed better synchrony between barley and soybean meal, as the higher rate of degradation of orange pulp than soybean meal was responsible for the greatest NH_3_-N in the liquor content and the greater fat added to the SH diet reduced the microbial fermentation [28]. Fat mobilization to meet energy requirements was found, thus TE_fat_ shown in Table 6 was -40.60 and -45 kJ/ kg of BW^0.75^ for OP and SH, respectively. High rates of fat mobilization led to markedly increased (P < 0.05) plasma concentrations of NEFA, BHBA and accumulation of triglycerides. These differences were found between OP and others, but not between OP and SH. Moreover, plasma glucose was greater in BRL than OP. During negative energy balance, blood glucose concentration is low and body fat is mobilized and transported as NEFA to several organs, particularly to the liver, where these FA are oxidized to produce energy. Excessive amounts of NEFA released during body fat mobilization are transferred to the milk. The major NEFA released are palmitic acid (C16:0), stearic acid (C18:0) and oleic acid (C18:1n9c), and the elevated concentrations in milk fat of those FA were identified as valuable early warning biomarkers for negative energy balance [39]. Greater (P < 0.05) concentration of palmitic acid (C16:0), heptadecanoic acid (C17:0) and cis vaccenic acid (C18:1n7) were found in OP and SH than BRL, and the highest value of oleic acid was found in SH diet.

**Table 11:** Metabolites in milk, urine and plasma of Murciano-Granadina goats (n = 12) during midlactation by type of diet and period.

	Diet^1^				*P-Value*
Item	BRL	OP	SH	SEM^2^	
Milk					
Urea_milk_, mg/L	419.6^a^	294.5^b^	393.8^a^	28.10	0.037
Urine					
Total protein, mg/L	147.5^b^	205.0^a^	103.3^b^	15.34	0.014
Urea, mg/L	16012.5^a^	10500.0^b^	9916.7^c^	111.91	0.046
Plasma					
Glucose, mg/L	510.0^a^	390.0^b^	416.7^a^^b^	42.29	0.038
NEFA^3^, mM/L	0.91^b^	1.48^a^	0.79^b^	0.121	0.017
Beta-Hydroxybutyrate, mM/L	1.11^b^	3.27^a^	0.84^b^	0.447	0.010
Ketone bodies, mM/L	1.27^b^	3.38^a^	0.94^b^	0.462	0.010
Triglycerides, mg/L	37.5^b^	80.0^a^	60.0a^b^	13.55	0.045
Urea, mg/L	389.0^a^	200.0^b^	330.0^a^	22.24	0.042

^a,b,c^Means within a row with different superscripts differ (P < 0.05).

^1^BRL = barley; OP = orange pulp; SH = soybean hulls.

^2^SEM = standard error of the mean.

^3^NEFA = non-esterified fatty acids.

Thus, our observations of the BRL diet suggest that glucogenic nutrients stimulate body fat deposition and the partitioning of ME into body tissues and milk (most of the HPx derived from OXCHO: 59.2%). The other two diets were lipogenic, and lipogenic nutrients originate either from fibre or dietary fat, or from body reserves [49]. Fat mobilization was found in OP and SH, although greater OXF (76.6%) was found in SH than OP (49.2%). Therefore, BRL behaved as a glucogenic diet, SH as a lipogenic diet and OP as intermediate, but due to possible asynchrony between protein degradation and type of carbohydrate, the NH_3_-N increased in OP and SH and fat mobilization increased to meet energy demand and milk performance.

## Conclusions

This paper provides data on energy partitioning, substrate oxidation, carbon and nitrogen balances, methane emissions and milk performance in Murciano-Granadina goats during midlactation fed mixed diets. Replacing 59% of barley in the diet by dry orange pulp or soybean hulls did not affect milk yield (2.16 kg/d) and no effect was found for chemical composition. The higher starch diet (BRL diet) resulted in positive energy balance and higher fibrous diets showed fat mobilization. On the other hand, the difference in degradation rate between orange pulp and soybean meal (OP diet) and high fat added to the SH diet appear to have affected ruminal fermentation, with greater NH_3_-N for the OP and SH diets. The OP and SH treatments (lower pentadecanoic acid (C15:0) and higher heptadecanoic acid (C17:0) content in the milk of goats fed fibrous by-product) suggested a negative impact of these diets on rumen bacterial fermentative activity and fat mobilization. Replacement of cereal grain with fibrous by-products did not increase methane emissions (57.4 L/goat per day, on average). Therefore, lactating goats could utilize dry orange pulp and soybean hulls diets with no detrimental effect on milk performance, although attention should be paid to fat added to the diet and the synchrony between the type of fibre and the source of protein used.
